# Phantom validation of 17 and 11 heartbeat MOLLI T1 mapping sequence at 3T

**DOI:** 10.1186/1532-429X-13-S1-P10

**Published:** 2011-02-02

**Authors:** Songtao Liu, Justin Huang, Martin Ugander, Christopher Sibley, Abiola Oki, Jing Han, Andreas Greiser, Daniel R Messroghli, Peter Kellman, Andrew E Arai, David A Bluemke

**Affiliations:** 1 NIH Clinical Center, Bethesda, MD, USA; 2 NHLBI, Bethesda, MD, USA; 3 FDA, Rockville, MD, USA; 4 Siemens AG, Erlangen, Germany; 5 Franz-Volhard-Klinik Charité, Berlin, Germany

## Background and objective

The Modified Look-Locker Inversion-Recovery (MOLLI) sequence (Messroghli, et al, JMRI, 2007) was optimized for myocardial T1 mapping on 1.5T. However, little data exists on T1 mapping at 3T. The standard MOLLI sequence uses three inversion-recovery blocks to acquire 11 images over 17 heartbeats (HB). The long breath hold could limit its clinical application in patients with cardiorespiratory compromise. A new 11 HB MOLLI protocol with two inversion-recovery blocks was introduced recently, which reduces scan time by 35%. This aim of this study is to verify both 17 HB and 11 HB MOLLI sequences at 3T.

## Methods

Agarose gel-cupric sulfate phantoms were scanned using both 17 HB and 11 HB MOLLI sequence at 3T (Verio, Siemens) with simulated heart rates from 40-110 beats per minute (bpm). An inversion-recovery spin-echo (IR-SE) sequence with TR of 10sec, TI varying from 10ms to 2500ms was used in the same configuration. The phantoms were also scanned using a spin-echo sequence with varying TE for T2 quantification. T1 maps were generated by MRMap (Messroghli, BMC Medical Imaging, 2010) and a region of interest inside each phantom was measured by ImageJ. Results were compared to each other and to IR-SE, which served as the reference standard. The phantoms studied had T1 times that ranged from 174-2660ms and T2 times ranging from 51-188ms

## Results

T1 estimates by both 17HB and 11HB MOLLI matched measurements by IR-SE up to ~500ms, but underestimated longer T1 times. This underestimation increased for higher heart rates (see figure). After correction, the difference between 17HB MOLLI and IR-SE was reduced to -0.8+/-2.3%, and difference between 11HB MOLLI and IR-SE was -0.4%+/-2.2%. The mean difference between 17HB MOLLI and 11HB MOLLI was 0.4+/-1.2% at 60 bpm, and this was similar at other heart rates.

**Figure 1:**
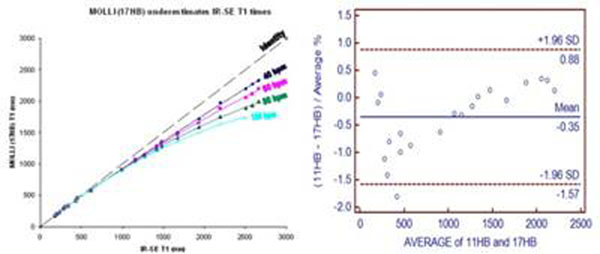
LEFT: 17HB MOLLI sequences progressively underestimated T1 compared to IR-SE in phantoms as T1 and heart rate increased. RIGHT: Bland-Altman plot compares 17HB MOLLI and 11HB MOLLI at 60 bpm**.**

## Conclusions

T1 mapping by MOLLI is feasible and accurate at 3T. Calibrated heart rate correction is necessary for T1 times longer than 500ms. Within our sample, 11HB MOLLI was an equally accurate estimator of IR-SE compared to 17HB MOLLI after correction was applied. Based on this, 17HB MOLLI and 11HB MOLLI can be used interchangeably.

